# Lignin Intermediates on Palladium: Insights into Keto‐Enol Tautomerization from Theoretical Modelling

**DOI:** 10.1002/cssc.202001560

**Published:** 2020-11-24

**Authors:** Ageo Meier de Andrade, Pemikar Srifa, Peter Broqvist, Kersti Hermansson

**Affiliations:** ^1^ Department of Chemistry-Ångström Uppsala University Box 538 75121 Uppsala Sweden

**Keywords:** density functional theory, energy decomposition, heterogeneous catalysis, keto-enol tautomerism, lignin depolymerization.

## Abstract

It has been suggested in the literature that keto‐to‐enol tautomerization plays a vital role for lignin fragmentation under mild conditions. On the other hand, previous modelling has shown that the adsorbed keto form is more stable than enol on the Pd(111) catalyst. The current density functional theory study of lignin model molecules shows that, in the gas‐phase, keto is more stable than enol, but on the Pd surface, we find enol conformers that are at least as stable as keto. This supports the experimental result that the favourable reaction pathway for lignin depolymerization involves keto‐enol tautomerization. An energy decomposition analysis gives insights concerning the origin of the fine energy balance between the keto and enol forms, where the molecule–surface interaction (−7 eV) and the molecular strain energy (+3 eV) are the main contributors to the adsorption energy.

## Graphical abstract

**It's a fine balance**: Experiments have suggested that keto‐enol tautomerism constitutes one important step in lignin's depolymerization reaction under mild conditions. In the gas‐phase, the keto form is more stable than the enol form, but the presented DFT study shows that, on the surface of a Pd catalyst, the enol conformers can be at least as stable as the keto conformers, supporting experiments.

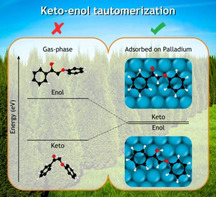

## Introduction

The context of this study is lignin valorisation through depolymerisation. The protective power of lignin in plant cell walls originates from the complex polymeric structure of lignin with its characteristic functional groups and strong linkages. These linkages are responsible for lignin‘s high resistance to chemical depolymerization (see, for example, the review article in Ref. [1], and references therein). The most common linkage is a β‐aryl ether bond, known as the β‐O‐4′ linkage; it represents 40–60 % of all linkage structures in lignin.^[2]^ Once depolymerized, the product molecules represent a high added value towards commercially relevant chemicals.[^3^, ^4^]

**Scheme 1:**
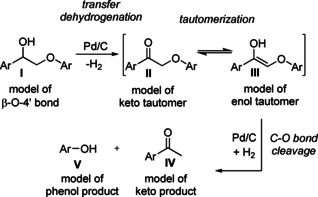
The Pd/C‐catalysed redox neutral cleavage of model **I** to products **IV** and **V**, via an initial transfer dehydrogenation to generate the intermediate tautomers **II** and **III**. Molecule **I** is 2‐phenoxy‐1‐phenylethan‐1‐ol, **II** is 2‐phenoxy‐1‐phenylethan‐1‐one, and **III** is (*Z*)‐2‐phenoxy‐1‐phenylethen‐1‐ol.

Noble metals such as palladium are known to catalyse the lignin depolymerization and promote selective hydrogenolysis towards aromatic monomers with a satisfactory yield at high temperature and high pressure.^[1]^ For mild reaction conditions, however, the situation is more challenging and much effort has been spent to optimize the reaction conditions with Pd as a catalyst, and to propose a reaction mechanism for the cleavage of the β‐O‐4′ linkage.^[5]^ The reaction mechanism occurring on the Pd surface is still under debate and at least two different routes have been proposed. Thus Zhou et al.^[6]^ suggested that for mild conditions (at 160 °C), the initial alcohol substrate, labelled (**I**) in Scheme 1, undergoes β‐O‐4′ hydrogenolysis with no keto‐enol tautomerism involved, while Galkin et al.^[5]^ proposed that the keto tautomer is formed after the first dehydrogenation step **(I)** to **(II)**, which is then followed by a keto‐to‐enol tautomerization in step **II** to **III**, and subsequent cleavage to **IV** and **V** (all at 80 °C). A tautomerization mechanism was (partly) supported by the comprehensive theoretical‐experimental study of Lu et al.,^[7]^ where the experiments (at 200 °C) demonstrated that tautomerization is likely to be involved in the reaction towards lignin cleavage. However, the theoretical calculations in the same paper found the adsorbed enol conformer to be less stable than the keto form (by 0.16 eV, i.e., 3.7 kcal/mol), which would impede the keto‐enol transformation. The first aim of the current study is to explore whether it is possible to identify an enol conformer that is in fact more similar in energy to, or lower than, the most stable keto form on the Pd catalyst surface.

We will discuss the subtle balance that determines the relative stabilities of the keto and enol tautomer forms within the chain of reactions shown in Scheme 1. To do so we will make use of density functional theory (DFT) calculations of a model lignin molecule on a model Pd(111) catalyst surface. A considerable number of DFT studies of lignin model systems have been published, involving aromatic molecules both in the gas‐phase (see, for example, Refs. [8–10]) and on model metal catalysts (see, for example, Refs. [11–13]). They provide a valuable context and reference frame for our study.

In addition to yielding structures and mechanisms of great detail, DFT calculations allow us to perform computer experiments for sub‐systems of our target system. Such so‐called decomposition schemes can be criticized for being artificial by construction, but they allow us to pinpoint one effect at a time within a complex scenario and decipher the respective roles of different contributing factors, yielding valuable new insight. Here, we will construct such a scheme to analyse different contributions to the stabilities of the enol and keto forms on the Pd surface. The second aim of the paper is to use the unique features of theoretical calculations to shed light on such structural and bonding features that stabilize the enol and keto forms on the Pd(111) surface.

The paper is organized as follows. The Computational Methods section describes the chemical systems, the electronic structure calculations, and the property calculations. The first aim listed above is addressed in the first two subsections of Results and Discussion, and the second aim is addressed in the two subsections thereafter.

## Computational Methods

### Model systems

The models chosen for the lignin molecules in this work were the 2‐phenoxy‐1‐phenylethanone (**II** in Scheme 1) and its tautomer enol form (*Z*)‐2‐phenoxy‐1‐phenylethen‐1‐ol (**III** in Scheme 1); see figures at the beginning of the Results and Discussion section. The molecules were placed on our model catalyst surface, namely Pd(111), which is the most stable extended Pd surface and it also tends to be the most abundant face on Pd nanoparticles.[^14^, ^15^]

Each model lignin/Pd(111) system (alternatively called interface systems in this paper) was constructed from a two‐dimensionally periodic Pd slab repeated periodically in the third direction, normal to the surfaces, with a 15 Å wide vacuum gap between the slabs. The Pd part was built from the optimized lattice constants of Pd bulk at the PBE‐D3 level (*a=*3.883 Å; the experimental low‐temperature bulk cell parameter of Pd is 3.8810 Å^[16]^) and contains four Pd layers, where the bottom two were kept fixed at the bulk structure. The molecule (keto or enol) was placed on one face of the Pd slab, with the phenyl rings lying essentially flat on the surface, and they remained so throughout the optimization. A number of different initial locations were explored for both keto and enol, as described in the second sub‐section of Results and Discussion. Five different sites were tested for each. We label these systems **Keto‐A**, …, **Keto‐E**, and **Enol‐A**, …, **Enol‐E**, in the order of stability we found, with A being the most stable.

Molecules **II** and **III** in principle fit well in a Pd $\left(6\times 2\sqrt{3}\right)$ supercell. This is the supercell that was used in our calculations. It is an orthorhombic cell with dimensions 16.473 Å and 9.510 Å (based on our optimized Pd bulk cell parameter).

Reference calculations for the keto and enol conformers in the gas‐phase were performed using a periodic cell with dimensions 20×20×20 Å^3^, and the other settings were consistent with the molecule/Pd calculations.

### Electronic structure calculations

We performed plane‐wave periodic DFT calculations with the PBE exchange‐correlation functional of Perdew et al.[^17^, ^18^] with D3 dispersion corrections according to Grimme et al.^[19]^ The Vienna ab initio simulation package (VASP)[^20^, ^21^, ^22^, ^23^] with a plane‐wave kinetic energy cutoff of 400 eV and pseudopotentials of the Projected Augmented wave (PAW) type developed by Blöchl^[24]^ and Kresse and Joubert^[25]^ were used. For H, O, C, and Pd, the 1s^1^, 2s^2^2p^4^, 2s^2^2p^2^ and 5s^0^4d^10^ electrons, respectively, were treated as valence electrons. Gaussian smearing with a broadening of 0.2 eV was applied to improve the electronic convergence.

All atomic positions were optimized (using the conjugate‐gradient algorithm) for all systems, except that the bottom two Pd layers in the Pd slab were always kept fixed at the bulk positions, as mentioned above. The optimization was pursued until the force on each atom was less than 0.01 eV/Å with a convergence criterion of 10^−5^ eV for the total energy threshold.

In our production calculations, a (4×4×1) *k*‐point grid following the Monkhorst–Pack scheme was used for all slab models (interface as well as bare slab). The choice of this grid size was made after careful testing. Our total energies for the ten molecule/Pd systems explored here were calculated with a (4×4×1) *k*‐point grid, and were converged to within 0.04 eV with respect to our reference energies, which were obtained using a (20×20×1) grid. The adsorption energies were shifted systematically by −0.03 to −0.04 eV towards stronger adsorption when the grid was increased from (4×4×1) to (20×20×1).

### Properties

#### Relative stabilities and adsorption energies

The relative thermodynamic stabilities for all the interface structures were computed with respect to a common reference, namely the **Keto‐A** interface system, which is the overall most stable molecule/Pd system that we found. Thus, for each of our interface systems, *E*_rel_ was calculated according to [Eq. (1)]:

(1) $$E{}_{\mathrm{rel}}=E{}^{\mathrm{tot}}\left(\mathrm{molecule}/\mathrm{Pd}\;\mathrm{slab}\right)-E{}^{\mathrm{tot}}\left(Keto\text{-}A\right)$$

where *E* ^tot^(molecule/Pd slab) is the total energy of the optimized interface structure containing an adsorbed lignin model molecule per supercell.

The adsorption energy per adsorbed molecule on the Pd(111) surface was calculated from Equation 2:

(2) $$E{}_{\mathrm{ads}}=E{}^{\mathrm{tot}}\left(\mathrm{molecule}/\mathrm{Pd}\;\mathrm{slab}\right)-[E{}^{\mathrm{tot}}\left(\mathrm{Pd}\;\mathrm{slab}\right)+E{}^{\mathrm{tot}}\left(\mathrm{gas}\text{-}\mathrm{phase}\;\mathrm{molecule}\right)]$$

where *E* ^tot^(Pd slab) is the total energy of the optimized bare 4‐layer Pd(111) slab, and *E* ^tot^(gas‐phase molecule) the total energy of the optimized lignin model in the gas phase.

Herein, the *E*_rel_ and *E*_ads_ notations are often used together with the superscripts “ZPE‐corr” (ZPE=zero‐point energy) or “no‐ZPE‐corr” to highlight whether the ZPE‐correction (see the next section) was applied or not.

#### Zero‐point energy correction

The total energies and the adsorption energies were calculated with and without a zero‐point energy (ZPE) correction. We included only the molecular contribution to the ZPE after careful testing and after observing that the effect of including the vibrational contributions from the slab to the ZPE correction was negligible. Neglecting the slab contributions is in fact a common procedure in the literature. Overall, the ZPE difference between the molecule adsorbed on the surface and the corresponding gas‐phase molecule was here found to be −0.14±0.03 eV for all ten different interfaces. In summary, the ZPE corrections shift all interface total energies to less negative values and all adsorption energies to more negative values. Essentially the relative stabilities of the various interface systems are very little affected by the inclusion of ZPE corrections.

## Results and Discussion

### Stabilities of the gas‐phase keto and enol conformers

**Figure 1:**
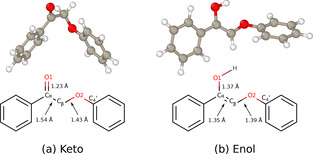
*Upper row*: Optimized gas‐phase structures of our lignin model molecules. a) The keto form (2‐phenoxy‐1‐phenylethanone, **II** in Scheme 1). b) The enol tautomer ((*Z*)‐2‐phenoxy‐1‐phenylethen‐1‐ol, **III** in Scheme 1). *Lower row*: The labelling schemes we use in this study. Here and in Figure 2, the Open Visualization Tool (OVITO) software^[31]^ was used to visualize the atomic structures.

As far as the gas‐phase species are concerned, the most stable structure of the keto molecule is a staggered conformer (Figure 1a) in which the oxygen of C_α_−O and the oxygen of the ether linkage C_β_−O−C_4_′ are located on opposite sides of the C_α_−C_β_ bond, in agreement with earlier computational results in the literature (see for example the detailed discussion in Ref. [26]). At the PBE‐D3 level, the keto structure is 0.21 eV more stable than the most stable enol structure, which is the (*Z*) conformer (Figure 1b). This relative order is in line with ample examples in the literature, experimental (see, for example, the results of Chiang et al.^[27]^) as well as theoretical,^[28]^ where the keto form of a gas‐phase molecule has been shown to be the most stable.

In the keto form of our molecule, the two phenyl rings are close to perpendicular to each other with a 95° angle between them. The enol structure has an intramolecular O⋅ ⋅ ⋅O distance of 2.710 Å which is typical of a medium‐long hydrogen bond. However, the O−H⋅ ⋅ ⋅O angle is as small as 113.5°, that is, the bond is very far from being linear, and the H⋅ ⋅ ⋅O distance is very long (2.17 Å); both these quantities are well beyond what is normally considered indicative of H‐bonding (see for example some of the comprehensive reviews on H‐bonding[^29^, ^30^]). Thus, this interaction is weak and should perhaps not even be considered to be a hydrogen bond.

### In search of stable keto and enol conformers on the Pd(111) surface

Next, we adsorbed the molecules on the Pd(111) surface, where they form a 2D periodic molecular layer held in place by the molecule‐surface interactions. The molecules were placed in various locations over the Pd(111) surface with respect to the location of both the two phenyl rings and the C_α_−C_β_ bond. During the geometry optimizations, the phenyl rings always remained firmly anchored on the surface at essentially their initial locations.

Based on earlier results in the literature, we avoided placing the phenyl rings such that their centres would fall atop a metal atom. In Ref. [32], for example, twenty‐seven different single‐ring aromatic molecules were placed on the Pt(111) surface (which is isostructural with Pd(111)), and PBE−D3 calculations showed that none of the molecules preferred to adsorb with the ring center atop a metal atom. Instead, a “bridge 30°” site was preferred, where bridge means that the centre of the aromatic ring crosses a Pt−Pt bond, and 30° means that the angle between the C_α_−C_β_ bond relative to the close‐packed metal‐metal bond is (approximately) 30°. Earlier, Morin et al.^[33]^ also found the bridge 30° orientation to be favourable for benzene adsorption on a number of metal surfaces investigated. For the Pd(111) surface explicitly, Lu et al.^[7]^ placed a phenol molecule in eight different positions on the surface, both at atop and bridge sites as well as above face‐centred cubic (fcc) and hexagonal close‐packed (hcp) hollow sites, and for each of these they tested a 0° and a 30° orientation of the C_α_−C_β_ bond. As in previous studies, the bridge 30° site was found to be the most favourable. However, the most stable adsorption site for aromatic rings on metallic surfaces is still under debate as demonstrated by the recent work of Treanor et al.^[34]^ who found the hollow hcp site to be (slightly) favourable on Rh(111).

**Figure 2:**
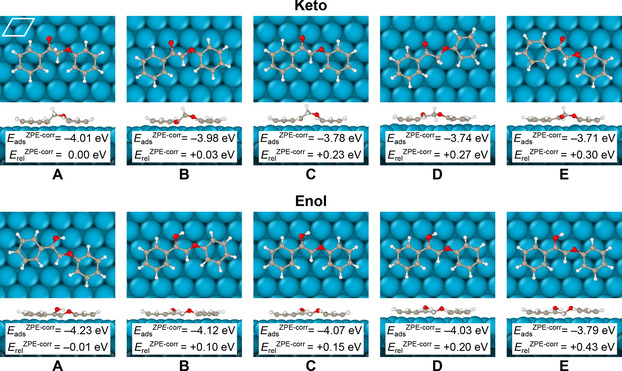
The ten optimized molecule/Pd(111) structures discussed herein, displayed as top and side views. In each row, the structures are ordered from the most stable (left) to the least stable (right). The relative stabilities (with **Keto‐A** as the reference) and the adsorption energies are also given in Table 1. The primitive surface unit cell for Pd(111) is shown in the upper left corner in the first panel.

The dominance of the bridge 30° site for the single‐ring molecules guided Lu et al. in their placement of lignin model derivatives with two phenyl rings on the Pd(111) surface in Ref. [7]: the phenyl groups were placed in bridge 30°‐bridge 30° conformations in the same Pd row. Here, and in the following, “the same row” means that the vector between the midpoints of the two phenyl rings is parallel to one of the primitive surface cell axes of the underlying Pd(111) substrate (the primitive surface cell is indicated in the first panel of Figure 2). In our study we additionally allow for adsorption over hollow sites and placement of the phenyl rings in different Pd rows.

Our resulting local minima structures are displayed in Figure 2. For each row, the structures are labelled A, B, C, D and E in order of stability, where A is the most stable. The labels **Keto‐A**, …, **Keto‐E**, **Enol‐A**, …, **Enol‐E** thus stand for the full molecule/Pd slab systems as shown in Figure 2.

Stability data and structural data are listed in Table 1. In summary, we find that

**Table 1:** Systems studied and key results: relative total energies, adsorption energies and structural parameters. 1^st^ column: the molecule/slab systems, 2^nd^ column: the relative energies of all interface systems referred to the **Keto‐A** conformer. 3^rd^ and 4^th^ columns: the adsorption energy, with and without ZPE‐correction respectively. 5^th^ and 6^th^ columns: structural parameters of the linkage regions. 7^th^ and 8^th^ columns: selected molecule‐surface distances. 9^th^ column: the locations of the adsorbed molecule‘s phenyl/phenoxy rings with respect to the underlying Pd atom(s). Energy and structural data for the optimized gas‐phase conformers are listed in the footnote and in the table, respectively. The atom labels (C_α_, C_β_, and O1) are given in Figure 1.

System	*E*_rel_^ZPE−corr^ [eV] [Eq. (1)]	*E*_ads_^ZPE−corr^ [eV] [Eq. (2)]	*E*_ads_^no−ZPE−corr^ [eV]	*R*(C_α_−C_β_) [Å]	*R*(C_α_−O1) [Å]	*R*(C_α_−Pd), *R*(C_β_−Pd) [Å]	R(O1−Pd) [Å]	Ring adsorption sites (phenyl, phenoxy)
*Keto*								
Keto (g)^[a]^				1.539	1.232			
**Keto‐A** ^[b]^	0.000	−4.008	−3.874	1.532	1.321	2.193, 3.035	2.137	(bri 30, bri 30)
**Keto‐B**	+0.031	−3.977	−3.837	1.534	1.320	2.184, 3.005	2.116	(bri 30, bri 30)
**Keto‐C**	+0.228	−3.780	−3.673	1.531	1.251	2.535, 3.434	2.892	(bri 30, bri 30)
**Keto‐D**	+0.271	−3.737	−3.606	1.545	1.237	2.672, 3.100	3.214	(bri 30, hollow)
**Keto‐E**	+0.302	−3.706	−3.576	1.542	1.233	3.033, 3.019	3.175	(hollow, bri 30)
								
*Enol*								
Enol (g)^[a]^				1.352	1.375			
**Enol‐A** ^[b]^	−0.013	−4.234	−4.084	1.482	1.372	2.249, 2.053	2.874	(hollow, bri 30)
**Enol‐B**	+0.100	−4.121	−3.968	1.418	1.360	2.387, 2.148	2.934	(bri 30, bri 0)
**Enol‐C**	+0.149	−4.072	−3.932	1.405	1.355	2.509, 2.168	3.025	(bri 30, bri 30)
**Enol‐D**	+0.195	−4.026	−3.861	1.427	1.365	2.265, 2.167	3.095	(bri 30, hollow)
**Enol‐E**	+0.434	−3.787	−3.627	1.464	1.361	2.293, 2.132	3.140	(bri 30, hollow)

[a] Keto(g) is 0.213 eV more stable than the Enol(g) conformer (ZPE‐corrected values). [b] The labels **Keto‐A**, …, **Keto‐E**, **Enol‐A**, …, **Enol‐E** stand for the full molecule/Pd slab systems as shown in Figure 2.

- **Enol‐A** and **Keto‐A** are (approximately) equally stable. This will have consequences for the interpretation of the keto‐enol tautomerization, as elaborated below.
- **Enol‐A** is not a bridge 30°−bridge 30° structure (see details in Table 1), and it extends over different rows.
- We retrieve the keto and enol structures reported in Ref. [7]. They correspond to our **Keto‐A** and **Enol‐C** structures, i.e., here we find two more stable enol structures.
- We find an alternative keto conformer, **Keto‐B**, that is very similar in stability to **Keto‐A**; this is a useful reminder of the multitude of local minima that will exist.

It is interesting to note that others have also found lignin fragment structures with two phenyl rings that span over several metal rows, such as the recent work by Phongpreecha et al.^[11]^ dealing with lignin models adsorbed on Ni(111) and Cu(111).

Next let us elaborate on the chemistry implied by point (i) in the 4‐point list above. We note that the most stable adsorbed enol structure in the calculations of Lu et al.^[7]^ was found to lie ∼0.16 eV above the most stable keto structure (labelled 2e in their paper and **Keto‐A** by us). In their reaction network, the keto structure has two alternative pathways: either dehydrogenation of H_β_ (bonded to C_β_), or tautomerization into enol (their 2 f) followed by dehydrogenation from the O−H group. Lu et al. also performed experiments under favorable conditions for keto‐enol tautomerization, and indeed found an enhanced yield of depolymerized product; they concluded that tautomerization is “likely involved in the reaction mechanism”. Solely from a thermodynamic point of view, however, an energy difference as large as 0.16 eV, as found in [7], makes this scenario, and the formation of the enol form on the surface of the catalyst, somewhat unlikely. On the other hand, if we now consider our **Enol‐A** conformer instead of **Enol‐C** (their 2 f) the picture changes. Leaning against the Bell–Evans–Polanyi principle our new result with the adsorbed enol and keto forms being very similar in magnitude suggests that the reaction pathway involving keto‐enol tautomerization is not only in competition with the keto dehydrogenation but might well be the dominant reaction pathway.

### Adsorption energy decomposition scheme

In the two previous sections, we analysed the relative stabilities of our molecule/Pd systems by means of their total energies. We will now highlight the very interplay between the molecules and the Pd slab by means of the adsorption energies.

*E*_ads_ is roughly 4 eV in all cases examined (Figure 2, Table 1). The molecule‐slab interaction is dominated by the phenyl rings; replacing them by methyl groups leads to adsorption energies that are approximately four times smaller judged from our results where methyl groups instead of phenyl rings were used.^[26]^

The adsorption energy is the result of a number of contributions. Those contributions can be discussed in terms of the traditional chemical and physical bonding types (polarisation, charge‐transfer, H‐bonding etc.) but here we will use a more pragmatic approach. For each optimized molecule/slab structure, the adsorption energy will be partitioned into four components, Δ*E*_1_+Δ*E*_2_+Δ*E*_3_+Δ*E*_4_, which describe sub‐reactions of the adsorption process according to:

$$\Delta E{}_{1}:\;\mathrm{Optimized}\;\mathrm{molecule}\left(g\right)=>\mathrm{Distorted}\;\mathrm{molecule}\left(g\right)\;$$

$$\Delta E{}_{2}:\;\mathrm{Optimized}\;\mathrm{clean}\;\mathrm{slab}=>\mathrm{Distorted}\;\mathrm{clean}\;\mathrm{slab}$$

$$\Delta E{}_{3}:\;\mathrm{Distorted}\;\mathrm{molecule}\left(g\right)=>\;\mathrm{Molecular}\;\mathrm{periodic}\;\mathrm{layer}$$

$$\Delta E{}_{4}:\;\mathrm{Molecular}\;\mathrm{periodic}\;\mathrm{layer}+\mathrm{distorted}\;\mathrm{clean}\;\mathrm{slab}=>\mathrm{Optimized}\;\mathrm{molecule}/\mathrm{slab}$$

**Figure 3:**
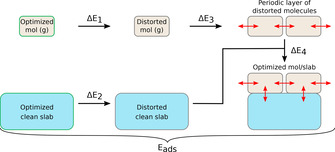
Graphical representation of the energy decomposition scheme used in this study and described in the text.

“Distorted molecule” means that its geometry is taken to be the same as the geometry that it has in the full optimized molecule/slab system, and analogously for the “distorted clean slab”. The adsorption process and the entailing energy components are illustrated in Figure 3. The sum of the four terms is equal to *E*_ads_ as defined in Equation (2), that is, we have

(3) $$E{}_{\mathrm{ads}}=\Delta E{}_{1}+\Delta E{}_{2}+\Delta E{}_{3}+\Delta E{}_{4}$$

Equation (3) is an identity, and we simply make use of the fact that enthalpy is a state function, that is, the total enthalpy change for a process (adsorption in our case) is independent of the path taken between them. This kind of exercise to decipher the energy contributions to a complex process is a well‐established approach in the theoretical literature; a few examples in the field of lignin model systems are given in Refs. [33,35,36].

**Figure 4:**
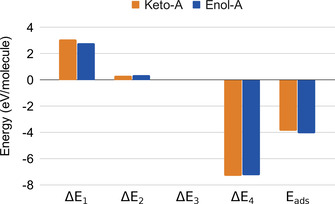
Energy values resulting from the energy decomposition scheme applied to the adsorption energies of **Keto‐A** and **Enol‐A**.

The results for the two most stable conformers of the enol and keto forms are given in Table 2, and results for the most stable conformer of each type are illustrated in Figure 4. Equation (3) is applicable to ZPE‐corrected and non‐ZPE‐corrected energies alike. In Table 2 we have used the latter type.

**Table 2:** Energy decomposition of *E*_ads_ for four of the interface structures studied herein. The energies given are non‐ZPE‐corrected energies. The definitions of Δ*E*_1_, Δ*E*_2_, Δ*E*_3_ and Δ*E*_4_ are given in the text.

Interface	Δ*E*_1_^[a]^ [eV]	Δ*E*_2_^[b]^ [eV]	Δ*E*_3_^[c]^ [eV]	Δ*E*_4_^[d]^ [eV]	*E*_ads_ [eV]
**Keto‐A**	+3.103	+0.341	−0.023	−7.295	−3.874
**Keto‐B**	+2.928	+0.352	−0.029	−7.088	−3.837
**Enol‐A**	+2.804	+0.380	−0.002	−7.266	−4.084
**Enol‐B**	+2.190	+0.348	−0.003	−6.503	−3.968

[a] Distortion energy of the molecule. [b] Distortion energy of the slab. [c] Intermolecular interaction. [d] Molecular film‐slab interaction.

First of all, we note that two energy contributions dominate *E*_ads_, namely the molecular distortion energy Δ*E*_1_, and Δ*E*_4_, which is the molecule‐slab interaction energy at the optimized molecule/slab geometry. The slab distortion energy Δ*E*_2_, on the other hand, is five to ten times smaller than Δ*E*_1_, and the intermolecular interaction within the free‐standing molecular layer (Δ*E*_3_) is very small.

The Δ*E*_3_ term is treacherous as it only monitors the interaction between neutral molecules. It involves the creation of a neutral free‐standing monolayer from the isolated molecules. Clearly the Δ*E*_3_ term will not capture any increased molecule‐molecule interaction arising for example as a consequence of charge transfer effects between the catalyst and the molecule.

Δ*E*_4_, on the other hand, involves the full molecule/Pd system and thus takes into account whatever charge transfer has occurred in the final system. We have calculated net atomic charges using the DDEC6 method[^37^, ^38^] and found that, for the **Keto‐A** interface, in total 0.33 electrons is transferred from the molecule to the slab, 0.35 electrons in the case of **Keto‐B**, and 0.57 electrons for **Enol‐A**. Using a simple electrostatic model, this charge transfer will create a dipole across the interface (or rather an array of dipoles as our systems are periodic) which will lead to dipole‐dipole repulsion that was not present before the adsorption occurred. Indeed, the Δ*E*_4_ term should be seen as a descriptor of the molecule‐surface interaction plus any additional molecule‐molecule interaction arising from polarisation of the molecules and/or charge transfer between the molecules and the surface.

Let us now scrutinize the *E*_ads_ values for **Enol‐A** and **Enol‐B** on the last two lines in Table 2. *E*_ads_ differs by only 0.12 eV between **Enol‐A** and **Enol‐B**, but the two major individual components (Δ*E*_1_ and Δ*E*_4_) differ by much more between the two molecules. Δ*E*_1_ differs by +0.61 eV and Δ*E*_4_ by −0.76 eV, leading to a compensation as they have different signs, and the resulting *E*_ads_ values become quite similar. It turns out that on binding to the Pd slab, the C_α_−C_β_ bond in **Enol‐A** loses much of its double‐bond character and elongates by 0.13 Å, consistent with the large Δ*E*_1_ penalty for this conformer, while the distortion is much less drastic for **Enol‐B**. The energy decomposition thus reveals new energy features that are hidden within the net *E*_ads_ values.

As a second example of the use of the decomposition scheme let us compare **Keto‐A** and **Enol‐A**. Their adsorption energies differ by 0.21 eV, with enol being the most strongly bound (Table 2). One might suspect that this difference primarily originates from the direct enol‐slab interaction Δ*E*_4_. However, the table shows the opposite: the Δ*E*_4_ values are almost the same for these two conformers. Instead it is mainly the difference in the distortion energies Δ*E*_1_ that lies behind the differences in adsorption energy. The keto molecule distorts more from its staggered gas‐phase structure (cf. Figure 1), and Δ*E*_1_ is 0.30 eV larger than that of enol.

### Bonding and correlations

In the previous section, we mentioned the modest charge transfer that takes place between the molecule and the metallic slab. Here we will return to the charges but also highlight some other bonding and structural aspects of the interaction between the molecule and the Pd surface. It is clear from Figure 2 that the linkage regions of **Keto‐A** and **Enol‐A** display significant structural differences.

**Figure 5:**
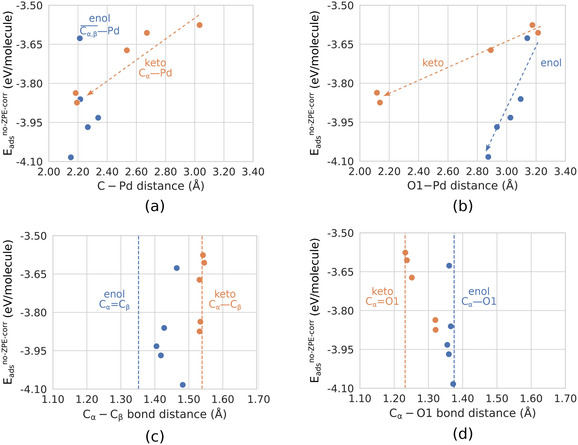
′*E*_ads_ vs. distance′ correlation plots for all ten interface structures. Data points for keto structures are colored orange, and enol data points are colored blue. Panels (a) and (b) show intermolecular distances, (c) and (d) show intramolecular distances. The dashed correlation arrows in (a) and (b) are just guides to the eye. The dashed vertical lines in (c) and (d) indicate the gas‐phase bond lengths. All energies and all distances are listed in Table 1. The atom labels (C_α_, C_β_, and O1) are given in Figure 1. See text for further details.

For all keto conformers in this work, C_α_ and O1 reside close to the surface, unlike C_β_, which points away from the surface. The linkage interacts with the Pd surface mainly through C_α_ and O1, which create two new bonds called C_α_−σ and O1−σ bonds. From this one might expect to find a correlation between the adsorption energy and the C_α_−Pd and O1−Pd distances. Indeed, Figure 5a and Figure 5b show that this is the case (orange points).

As for the intramolecular distances of the keto conformers, Figure 5c shows that in all cases the C_α_−C_β_ single bond length is virtually constant and the same as in the optimized gas‐phase molecule within less than 0.01 Å (cf. Table 1). However, as a consequence of the new C_α_−σ and O1−σ bonds, the C_α_−O1 bond length increases and displays some correlation with the *E*_ads_ energy (Figure 5d). The double bond character of C_α_−O1 approaches that of a single bond for large (negative) *E*_ads_ values and the molecule develops a positive net charge as electron density migrates from the C_α_−O1 double bond towards the C_α_−σ and O1−σ bonds with Pd. The positive molecular charge was mentioned already in the discussion of Δ*E*_4_.

Next, we go on to discussing the enol conformers (blue points). Here, C_α_ and C_β_ are the linkage atoms residing closest to the surface and, contrary to the keto cases, the O1 atom points away from the surface. In all the enol conformers, there is significant weakening (elongation) of the C_α_−C_β_ double bond compared to the gas phase (horizontal dashed line); see Figure 5c. The elongation of the bond is accompanied by the creation of a Pd−C_α_−C_β_−Pd moiety, often called a di−σ bond. Also, here the molecule develops a positive net atomic charge.

We calculated the C_α_−Pd and C_β_−Pd distances for the enol conformers, but observe no correlation with *E*_ads_, regardless of whether the energy is plotted against the C_α_−Pd distance, the C_β_−Pd distance, or the average of the two distances; the latter is the choice used in Figure 5a. On the other hand, we observe a correlation between the O1−Pd distance and *E*_ads_ (Figure 5b), which we did not expect as O1 is pointing away from the surface. Using more data points (conformers) or a more elaborate structural descriptor may shed insight here.

## Conclusions

Experimental results in the literature have suggested that the keto‐enol tautomerization is an important reaction step in the depolymerization of lignin. Earlier computational results in the literature showed that the keto form is more stable on the Pd surface than enol by 0.16 eV, which thermodynamically makes the reaction unlikely to occur. We have explored the bonding and interactions of keto and enol conformers of a lignin model molecule on Pd(111) using DFT. Considerable care was taken to achieve tight optimizations, include dispersion correction, explore the convergence of the *k*‐point grid and apply zero‐point energy corrections. Our main results are as follows.

(i) We found two new low‐energy enol conformers compared to earlier work. Our most stable enol conformer binds more strongly on the Pd(111) surface than the keto conformer: *E*_ads_(enol)=−4.23 eV and *E*_ads_(keto)=−4.01 eV. In the gas phase, on the other hand, the keto form is more stable than the enol form by 0.213 eV, but the difference between the adsorption energies of the enol and keto conformers is large enough to match, and even overcome, the larger gas‐phase stability of the keto molecule. The result is that when adsorbed on the Pd (catalyst) surface the keto and enol forms are very similar in stability. This result strengthens and highlights the likelihood of keto‐enol tautomerization as a key intermediate reaction step in the lignin fragmentation.

(ii) An energy decomposition scheme was applied to identify which are the most important contributions to the adsorption energies of the enol and keto molecules on the Pd metal. The most stable conformers of each kind have *E*_ads_ values which are roughly about −4 eV. This value is essentially a result of partial cancellation of two large contributions with different signs: the molecule–surface interaction Δ*E*_4_ (−7 eV) and the molecular strain energy Δ*E*_1_ (+3 eV). The Δ*E*_4_ term in the decomposition scheme should be interpreted as containing the effects of adhesion of the (neutral) film on the surface plus any energy contributions arising from polarisation and charge transfer processes occurring upon adhesion.

(iii) Keto conformers bind to the Pd surface through the C_α_ and O1 atoms, forming two new chemical bonds, namely C_α_−σ and O1−σ bonds. The enol–surface interaction results in a weakening of the C_α_−C_β_ bond as a result of the newly formed di−σ bond. These conclusions are supported by the *E*_ads_‐distance correlation results displayed in Figure 5.

## Conflict of interest

The authors declare no conflict of interest.
